# Regulation of Mammalian Mitochondrial Gene Expression: Recent Advances

**DOI:** 10.1016/j.tibs.2017.02.003

**Published:** 2017-08

**Authors:** Sarah F. Pearce, Pedro Rebelo-Guiomar, Aaron R. D’Souza, Christopher A. Powell, Lindsey Van Haute, Michal Minczuk

**Affiliations:** 1 Mitochondrial Genetics, Medical Research Council (MRC) Mitochondrial Biology Unit, Hills Road, Cambridge CB2 0XY, UK; 2 Graduate Program in Areas of Basic and Applied Biology (GABBA), University of Porto, Porto, Portugal

## Abstract

Perturbation of mitochondrial DNA (mtDNA) gene expression can lead to human pathologies. Therefore, a greater appreciation of the basic mechanisms of mitochondrial gene expression is desirable to understand the pathophysiology of associated disorders. Although the purpose of the mitochondrial gene expression machinery is to provide only 13 proteins of the oxidative phosphorylation (OxPhos) system, recent studies have revealed its remarkable and unexpected complexity. We review here the latest breakthroughs in our understanding of the post-transcriptional processes of mitochondrial gene expression, focusing on advances in analyzing the mitochondrial epitranscriptome, the role of mitochondrial RNA granules (MRGs), the benefits of recently obtained structures of the mitochondrial ribosome, and the coordination of mitochondrial and cytosolic translation to orchestrate the biogenesis of OxPhos complexes.

## Trends

The genetic system required for mitochondrial gene expression is housed within the mitochondrial matrix, with all the necessary RNAs being provided by transcription of the mtDNA itself.

Our understanding of the extent and nature of post-transcriptional modifications of mtRNA, the epitranscriptome, is rapidly expanding. Several required nucleus-encoded enzymes have recently been identified.

mtRNA maturation factors localize in distinct foci, termed mtRNA granules, with newly transcribed RNA. These foci may allow spatiotemporal control of mtRNA processing.

Recent high-resolution structures obtained via cryo-electron microscopy have rapidly advanced our understanding of the specialized adaptations of the mitochondrial ribosome.

Production of respiratory complexes requires tight coordination between the cytoplasmic and mitochondrial translation systems.

## Mitochondria and Their Genes

Mitochondria are dynamic organelles that are present in almost all eukaryotic cells and play a crucial role in several cellular pathways. Their most recognizable role is providing the cell with energy in the form of ATP via OxPhos. However, many other functions have been assigned to mitochondria, including the integration of metabolic pathways (such as the biosyntheses of heme, iron–sulfur clusters, and nucleotides), apoptosis, and reactive oxidative species (ROS) signaling.

The endosymbiotic theory proposes that mitochondria originated as free-living Alphaproteobacteria that were internalized by a pre-eukaryotic host cell, leading to the formation of the modern eukaryotic cell. In the course of evolution, the genome of the original alphaproteobacterial symbiont has undergone extensive reduction. The majority of its genes have either been lost, owing to redundancy, or transferred to the host nuclear genome. Furthermore, mitochondria have lost autonomy over their genome maintenance and expression to the host cell. Nonetheless, in almost all cases, eukaryotic mitochondria retain a minimal genome, of variable size and gene content, that is present in many copies within their matrix.

Human mitochondrial DNA (mtDNA) is a circular molecule of ∼16.5 kb which encodes a small subset of the structural polypeptide components required for OxPhos. These mRNAs are transcribed and then translated within the mitochondrial matrix by a dedicated, unique, and highly specialized machinery. The RNA components of the mitochondrial gene expression system, two mitochondrial ribosomal RNAs (mt-rRNAs) and 22 mt-tRNAs, are also encoded by mtDNA, whereas all other protein components are encoded by nuclear genes and imported into mitochondria from the cytosol. Based on published research 1, 2 and our unpublished data, we estimate that 250–300 nucleus-encoded proteins are dedicated to serve mitochondrial gene expression. This includes RNA polymerase and transcription factors, endonucleases for RNA precursor processing, aminoacyl-tRNA synthetases, RNA-modifying enzymes, the structural components and biogenesis factors for the mitochondrial ribosome, translation factors, and other auxiliary factors.

Many mutations in mtDNA that affect the expression of mitochondria-encoded OxPhos components are associated with human pathologies, collectively known as mitochondrial disease. Recent growing evidence also suggests that defects in the nuclear genes involved in mitochondrial gene expression are also one of the major cause of human mitochondrial disease 3, 4, 5. However, establishing the molecular details of how these defects contribute to pathogenicity and patient phenotypes constitutes a major challenge.

**Figure 1:**
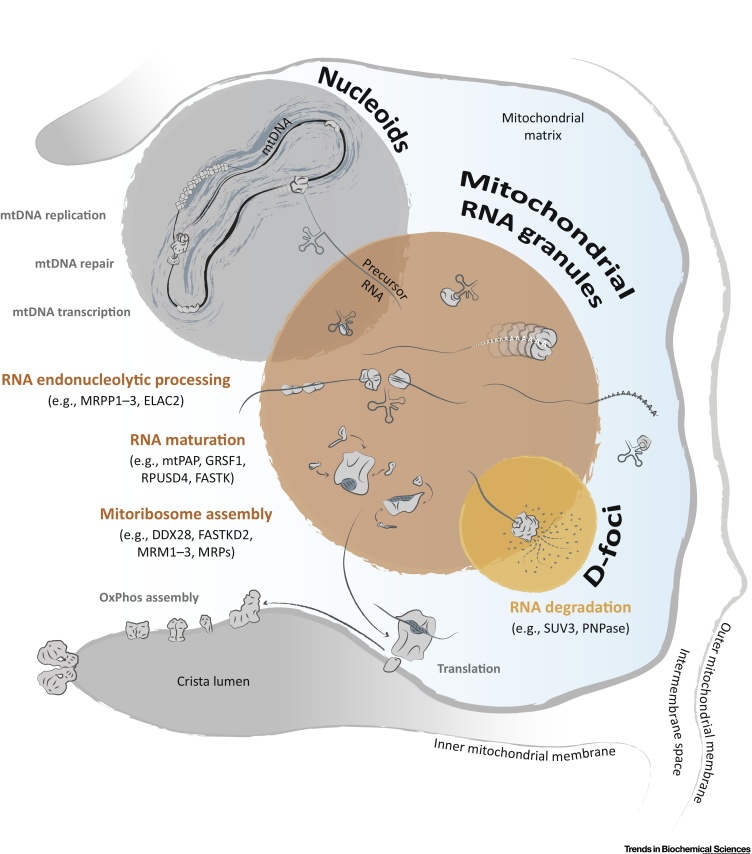
Mitochondrial Gene Maintenance and Expression: A Focus on Post-Transcriptional Processes. Proteins involved in mitochondrial gene maintenance and expression have been localized to focal nucleoprotein structures in the mitochondrial matrix. These foci can be classified according to their protein contents into nucleoids, which contain mitochondrial (mt)DNA and mtRNA granules (MRGs, represented in dark orange). Nucleoids and MRGs are present in close spatial proximity in microscope analyses. Nucleoids contribute mainly to the maintenance of the genetic material of mitochondria and the synthesis of RNA. Characterization of MRGs revealed the presence of a panoply of enzyme classes that perform diverse tasks necessary for the post-transcriptional expression of mitochondrial genes, from RNA maturation to mitoribosome assembly. However, it is currently not clear if all post-transcriptional steps of mitochondrial gene expression occur in MRGs. RNA degradation, the last stage of RNA metabolism, has also been postulated to occur in specialized foci, termed D-foci (shown in light orange), that contain the components of the mitochondrial degradosome, RNA helicase Suv3 and PNPase. A portion of D-foci have been found to colocalize with newly synthesized mtRNA, in a similar manner to MRGs, and may represent a subpopulation of MRGs participating in RNA processing or degradation mediated by the degradosome. Following maturation of all three classes of mtRNAs, mitoribosome assembly, and mt-tRNA aminoacylation, these molecules are now available for utilization in mitochondrial protein synthesis. Mitoribosomes synthesize 13 OxPhos proteins encoded by mt-mRNAs, and these subunits are co-translationally inserted into the inner mitochondrial membrane where they are incorporated into respiratory complexes together with nucleus-encoded OxPhos subunits. The stages of mitochondrial gene expression relevant for this review are indicated in orange.

Although mitochondrial gene expression requires a series of linked processes encompassing mtDNA repair, replication, transcription (reviewed in 6, 7), and mtRNA maturation (reviewed in [8]) through to translation (reviewed in [9]) and respiratory complex assembly (reviewed in [10]), this article will focus in particular on the most recent advances in our understanding of the post-transcriptional processes of mtDNA gene expression (Figure 1). In particular, we outline the recent identification of novel nucleus-encoded factors required for modification or maturation of mtRNAs, the characterization of mtRNA granules (MRGs) as sites for organization of mtRNA processing, our improved understanding of the structure and composition of the mammalian mitochondrial ribosome, and of the coordination required for concerted nuclear and mitochondrial gene expression.

## The Mitochondrial Epitranscriptome: Chemical RNA Modification for Gene Regulation

**Figure 2:**
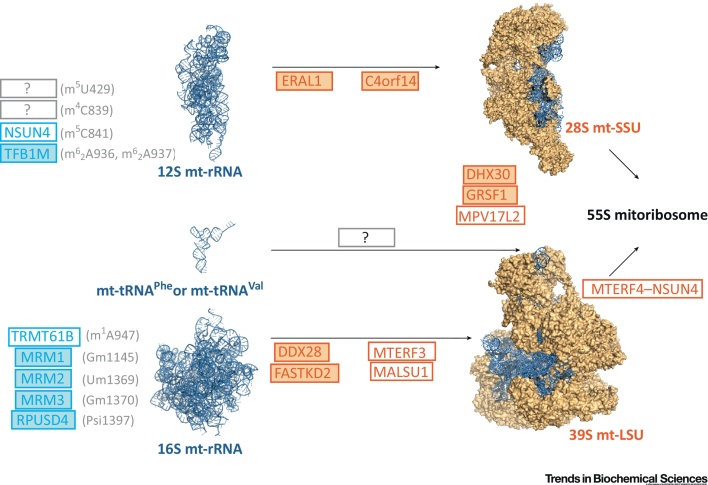
Known Players in Mammalian Mitochondrial Ribosomal Biogenesis. Following endonucleolytic cleavage of newly transcribed polycistronic mitochondrial RNA (mtRNA) transcripts, mitochondrial ribosomal RNAs (mt-rRNAs) must undergo a series of nucleotide modifications. There are five known modified residues in mammalian 12S mt-rRNA and five in 16S mt-rRNA. The exact residues and their modifications are indicated in brackets and relate to the position in human 12S or 16S mt-rRNA. Shown in blue are the enzyme factors that are known to modify the indicated positions on mt-rRNAs. Several methyltransferases have been identified which modify mt-rRNAs, with NSUN4 and TFB1M modifying 12S and TRMT61B, and MRM1, MRM2, and MRM3 modifying 16S, at the indicated positions. In addition, pseudouridine synthase RPUSD4 has been identified as enzyme that is likely to provide the Psi1397 modification. In addition to nucleotide modifiers, several further protein factors are required for assembly of mitochondrial ribosomal proteins (MRPs) into the mitochondrial large ribosomal subunit (mt-LSU), the mitochondrial ribosomal small subunit (mt-SSU), or to aid in the formation of the complete monosome. These accessory factors are shown in orange. Although the enzymatic role of some of these factors have been predicted or can be inferred from their bacterial homologs, such as the putative RNA helicases MDDX28 and DHX30, or the proposed RNA chaperone ERAL1, the precise roles of many of the indicated accessory factors in mitoribosome biogenesis have yet to be determined. ‘?’ indicates a known nucleotide modification or assembly step where the responsible factors are yet to be identified. Because MRPs have been found to copurify with the mitochondrial nucleoid and mtRNA granules (MRGs), early steps of mitoribosome assembly are likely to occur co-transcriptionally, in concert with mt-rRNA nucleotide modifications. The factors that have, thus far, been found to localize to MRGs are in solid color. Abbreviations: LSU, large subunit; PSI, pseudouridine; SSU, small subunit.

Control of the transcriptome in any biological system requires numerous post-transcriptional RNA modifications. In line with this, transcripts released from polycistronic mtRNA precursors are subject to enzymatic nucleotide modifications in mammalian mitochondria (Box 1). Early comprehensive biochemical work by Dubin *et al.* using hamster RNA identified a set of nine well-conserved chemical modifications to the small (12S) and the large (16S) mt-rRNAs (Figure 2) 104, 105, 106, 107. Most of these modifications have been confirmed to be present in the mt-rRNA of humans and other mammals, and several factors responsible for introducing these modifications have been identified 11, 12, 13, 14. In 12S mt-rRNA, TFB1M is responsible for nucleobase dimethylation of adenines A936 and A937 13, 15, whereas NSUN4 methylates cytidine at position 841 (human numbering) [14]. A group of closely related putative 2′-*O*-ribose methyltransferases (MRM1, MRM2/FtsJ2, and MRM3/RNMTL1) have been shown to be involved in modification of three nucleotide positions of the peptidyl transferase center of 16S mt-rRNA 11, 12 (Figure 2). Very recently, a 10th modification in mt-rRNA was identified (16S: m^1^A947), initially by analyzing RNA–DNA differences (RDDs) in RNA-Seq experiments, followed by biochemical confirmation and identification of TRMT61B as the enzyme responsible [16]. A systematic survey has revealed that ∼7% of all bovine mt-tRNA residues undergo post-transcriptional modification, with over 30 different modified mt-tRNA positions [17]. Several enzymes introducing these modifications have already been determined, but the list is far from complete (18, 19 for recent comprehensive reviews). The number of modified nucleotides in the mitochondrial non-coding RNA is generally greatly reduced compared to their cytoplasmic or bacterial counterparts [20]. For example, the bacterial large 23S rRNA harbors 25 modifications (∼1%), whereas only five modified nucleotides have been detected in human 16S mt-rRNA (∼0.3%). This suggests that the few mtRNA modifications that have been retained are of high functional importance. One of the reasons for the lower level of modifications on mtRNA could be the limitation of the number of nuclear DNA (nDNA)-encoded protein factors that must be imported into mitochondria. Alternatively, it could have resulted from an inability of mitochondria to adapt the small nucleolar (sno) RNA-guided modification mechanisms that operate in the cytoplasmic ribosome [21]. However, with only a few modified nucleotides, mammalian mitochondria appear to have found alternative solutions for the proper functioning of their tRNA and rRNA. This has been most strikingly demonstrated by the complete mapping of post-transcriptional modifications in the mt-tRNAs of *Bos taurus*, in which the very highly conserved m^5^U54 methylation is entirely absent [17]. The adaptive changes that have arisen to compensate for its loss, however, are currently unclear.

Box 1 **Key Steps in mtRNA Expression and Maturation**

The human mitochondrial genome, or mtDNA, encodes 13 polypeptides, 22 mitochondrial transfer RNAs (mt-tRNAs), and two ribosomal RNAs (mt-rRNAs). mtDNA transcription, therefore, provides all the RNA components required for translation of its own messenger RNAs (mt-mRNAs). Genes are spread across both the heavy (H) and light (L) strands of mtDNA. Instead of initiating at individual gene-specific promoters, transcription of mammalian mtDNA initiates from single promoters for H- and L-strand transcription, and progresses around almost the entire length of the genome.

A single-subunit RNA polymerase, POLRMT, is responsible for mtDNA transcription, and provides both the promoter-binding specificity and catalytic polymerase activity 86, 87. In addition, mitochondrial transcription factor A (TFAM), mitochondrial transcription factor B2 (TFB2M), and mitochondrial transcription elongation factor (TEFM) are required for efficient transcription *in vivo* 88, 89.

Because transcription results in polycistronic transcripts, the constituent mt-mRNAs, mt-tRNAs and mt-rRNAs must be released via endonucleolytic cleavage. In the majority of cases, human mt-mRNAs and mt-rRNAs are flanked at both the 5′ and 3′ ends by mt-tRNA genes, allowing their excision to release mt-mRNAs and mt-rRNAs via the ‘tRNA punctuation model’ [90]. This cleavage is performed by a mitochondria-specific, protein-only RNase P complex [91] and ELAC2 [92], which catalyze 5′ and 3′ endonucleolytic cleavage, respectively. There are, however, some exceptions where mt-tRNAs are not positioned at mt-mRNA junctions. Although the exact mechanisms for cleavage at such non-canonical junctions remain to be determined, recent studies have implicated FASTK, FASTKD4, and FASTKD5 as well as GRSF1 as factors involved in these processes 49, 64, 65, 70.

Following endonucleolytic processing, individual mt-mRNA, mt-rRNA, and mt-tRNA transcripts undergo post-transcriptional modifications. Several nucleotides in mt-rRNAs are modified to facilitate mitoribosome biogenesis and function before incorporation into the mitoribosome proteins (Figure 2). The only confirmed modification of mt-mRNAs is the addition of a 3′ poly(A) tail catalyzed by mitochondrial poly(A) polymerase (mtPAP) [93], with the exception of ND6 mt-mRNA which is not polyadenylated. Control of poly(A) tail length and RNA stability is achieved with the aid of the complex of LRPPRC–SLIRP 94, 95, 96 and the PDE12 poly(A)-specific exoribonuclease [97].

Finally, mt-tRNAs undergo extensive post-transcriptional nucleotide modification, in addition to 3′ CCA trinucleotide addition [18], before being aminoacylated with their cognate amino acid [98]. Following maturation of mt-RNAs and mitoribosomes, they are available for use in mitochondrial protein synthesis.

Alt-text: Box 1

### RNA Epigenetics: NSUN3 and ABH1 As New Players in Decoding Mitochondria-Specific Codons

One of the recently investigated unique features of mitochondrial gene expression concerns the post-transcriptional modification of mt-tRNA^Met^ and decoding of methionine codons. In the cytosol of eukaryotic cells there are two distinct types of tRNA^Met^ that are used for either translation initiation or elongation. However, as a consequence of the reductive evolution of mtDNA, metazoan mitochondrial transcriptomes contain a minimal set of tRNAs, where each mt-tRNA species usually decodes an increased number of codons. In line with this, human mtDNA contains only one gene encoding mitochondrial tRNA^Met^ that is used for both initiation and elongation stages of mitochondrial protein synthesis. However, in addition to decoding the universal AUG codons, this single mt-tRNA^Met^ also recognizes the unconventional AUA (which codes for isoleucine in the universal genetic code) during initiation and elongation 22, 23. In the human mitochondrial genome, 167 methionine residues are encoded by AUA, and only 40 use the standard AUG codon. Furthermore, mt-tRNA^Met^ can also decode AUU and AUC, but only as initiation codons (during elongation AUU and AUC are decoded as isoleucine, as in the standard genetic code). It has previously been shown *in vitro* that a 5-formyl modification of the cytosine residue in the wobble position of the anticodon (f^5^C34) in human mt-tRNA^Met^ allows decoding of AUG and the non-conventional AUN codons 23, 24. Even though mt-tRNA^Met^ f^5^C34 was first detected over 20 years ago [22], until recently the source of the formyl group and the enzyme(s) involved in generating this modification remained unknown.

Three independent studies have recently enabled identification of the sequential mechanism responsible for the f^5^C34 modification of the wobble cytosine in mt-tRNA^Met^ 25, 26, 27. Using a variety of different approaches, the three laboratories found that this nucleobase is initially modified with a methyl group (m^5^C) that is subsequently oxidized to a formyl group, generating f^5^C. The enzyme responsible for the methylation step has been identified as NSUN3, a mitochondria-targeted member of the Nol1/Nop2/Sun domain (NSUN) family of m^5^C RNA methyltransferases. Inactivation of NSUN3 resulted not only in reduced levels of m^5^C34 but also in decreased levels of f^5^C34. This observation proved that methylation constitutes an intermediate step in the pathway of formylation of the mt-tRNA^Met^ wobble cytosine. One of these three studies [25] has also identified the enzyme responsible for methyl- to formylcytosine conversion as ABH1 (also known as ALKBH1). ABH1 belongs to a family of Fe^2+^/α-ketoglutarate-dependent dioxygenases. Inactivation of either NSUN3 or ABH1 led to defects in mitochondrial translation. However, it is currently not clear whether the entire functional pool of mt-tRNA^Met^ is formylated at C34 *in vivo*, with the methylated form being merely an intermediate, or whether differentially modified mt-tRNA^Met^ pools are present in mitochondria. Because α-ketoglutarate is an important intermediate of the Krebs cycle, the levels of α-ketoglutarate could potentially influence methyl to formylcytosine conversion by ABH1 and, as such, regulate the m^5^C34/f^5^C34 ratio. It will be fascinating, therefore, to establish if alterations to the relative abundance of different RNA modifications could potentially participate in the regulation of mitochondrial translation as a response to changes in the metabolic state of the cell.

### RNA Pseudouridylation: A New Twist

Pseudouridine (Ψ), the most common modification in non-coding RNA, is a structural isomer of uridine and the only mass-silent RNA modification identified so far [28]. As do their cytoplasmic counterparts, mt-tRNAs contain pseudouridylated residues [17]. It has also been determined that mammalian 16S mt-rRNA contains at least one Ψ [29] (Figure 2). The roles of individual Ψs, the enzymes responsible for these modifications, and how they influence the structure and function of different mtRNA classes remain to be clarified. Of several conserved human Ψ synthases, only PUS1 has previously been characterized as an mt-tRNA modifying enzyme (positions 27 and 28) and has been linked to human disease [30]. However, a recent genome-wide CRISPR ‘death screen’ identified three previously uncharacterized, putative Ψ synthases (RPUSD3, RPUSD4, and TRUB2) as being essential for OxPhos [31]. Depletion of any of these genes results in decreased levels of 16S mt-rRNA and perturbs mitochondrial translation. Very recently, these three putative mitochondrial Ψ synthases were shown to localize to MRGs, the concept of which is discussed in greater detail below 32, 33. Furthermore, RPUSD4 is probably the enzyme responsible for pseudouridylation of position 1397 of 16S mt-rRNA [32]. This is consistent with a further study which demonstrated instability of 16S mt-rRNA upon RPUSD4 silencing [33]. However, further research will be necessary to determine the exact role of 16S mt-rRNA pseudouridylation, especially in the context of mitochondrial ribosome biogenesis (Figure 2 and see below).

A further high-throughput study that analyzed transcriptome-wide occurrence of Ψs indicated that, in addition to non-coding RNA, mt-mRNAs also contain this modification [34]. Although a more targeted validation of this finding is required, mt-mRNA pseudouridylation could emerge as an important regulatory mechanism; for example, by rewiring the genetic code via non-canonical base pairing in the ribosome decoding center. Characterizing the landscape, enzymology, and potential dynamic changes in pseudouridylation of the mitochondrial transcriptome is likely to be a subject of intense study in the near future.

As we describe above, the field has experienced rapid expansion in our knowledge of the modifications of mtRNAs, and of the nuclear factors that are responsible for these modifications (e.g., NSUN3, ALKBH1, Ψ synthases). However, further work will be necessary to fully characterize the mitochondrial epitranscriptome and the functional role for each RNA modification in the context of mt-tRNA biology and mitoribosome biogenesis.

## Mitoribosome Structure and Biogenesis

Recent developments in cryo-electron microscopy (cryo-EM) have transformed our access to 3D structures of protein complexes that have thus far been refractory to other methods. The near-atomic resolution structure of mammalian mitochondrial ribosomes (mitoribosomes) has recently been obtained, revealing several unexpected features 35, 36, 37, 38 (Box 2).

Box 2 **The Mammalian Mitochondrial Ribosome**

The mammalian mitochondrial ribosome (mitoribosome) is specialized for the translation of the 13 mitochondria-encoded components of the OxPhos system. The two subunits of the mitoribosome, the 28S small subunit (mt-SSU) and the 39S large subunit (mt-LSU), comprise a catalytic RNA – 12S mt-rRNA and 16S mt-rRNA, respectively – encased in protein subunits (MRPs).

The co-evolution of the mammalian mitoribosome along with the highly hydrophobic OxPhos subunits has resulted in drastic changes to the composition and architecture of the mitoribosome complex. Compared to bacterial, eukaryotic cytoplasmic ribosomes, and yeast mitoribosomes, the RNA component of the mammalian mitoribosome is about half the length, and this may be a consequence of reductive evolution of the mitochondrial genome [99]. To compensate for the severe reduction in rRNA size, the mitoribosome has recruited more protein subunits to stabilize the structure and compensate for loss of functional domains 37, 38. In contrast to the 55 ribosomal proteins in the bacterial ribosome, and the 70 proteins in the yeast mitoribosome, the mammalian mitoribosome consists of 80 protein subunits. In addition, many RNA–RNA interactions which bridge between the large and small subunits in the bacterial and eukaryotic cytoplasmic ribosomes have been replaced by protein–protein and protein–RNA bridges in the mammalian mitoribosome 37, 38.

The remodeling of the mitoribosome is particularly noticeable in the central protuberance (CP) region, which in bacterial, archaeal, and eukaryotic cytoplasmic ribosomes contains a 5S rRNA component. In the mammalian mitoribosome this has been replaced by an mtDNA-encoded tRNA. The identity of the tRNA assembled into the mitoribosomes varies depending on the organism. Both mt-tRNA^Val^ and mt-tRNA^Phe^ have been shown to be present in the CP 35, 36, 100. Both these mt-tRNAs are located adjacent to mt-rRNAs in the human mitochondrial genome, allowing co-transcriptional maturation and assembly into the mitoribosome. Moreover, mammalian mitoribosomes have acquired a GTP-hydrolyzing protein (mS29) and a pentatricopeptide repeat (PPR) protein (mS39), the functions of which are not fully understood [9].

The polypeptide exit tunnel of the mitoribosome has also become specialized for the translation of hydrophobic membrane proteins. The exit tunnel of the mitoribosome is positioned such that nascent polypeptide can be cotranslationally inserted into the membrane and the large subunit of the translating monosome is attached to the membrane by the C-terminal helix of mL45. Moreover, residues lining the tunnel surface are hydrophobic to regulate the rate at which of the newly synthesized polypeptide exits from the mitoribosome 35, 36.

Although the recently resolved cryo-EM structures have rapidly accelerated our understanding of the structural composition of the mitoribosome, they do have some limitations, warranting further study. During translation, mammalian mitoribosomes must be localized to the inner mitochondrial membrane, in association with the protein machinery required for membrane insertion of elongating polypeptides, which remains to be fully characterized. Therefore, further structural studies to address the exact nature of the interaction of actively translating mitoribosomes with the inner mitochondrial membrane would be highly advantageous.

Alt-text: Box 2

### Plasticity of Structural RNA Selection by the Mammalian Mitoribosome

Mammalian mitoribosomes differ from other ribosomes by the unexpected integration of a mitochondria-encoded tRNA as a structural element of the central protuberance (CP) region of the large subunit (mt-LSU). Interestingly, different mt-tRNAs have been reported to be present in human (mt-tRNA^Val^) and porcine (mt-tRNA^Phe^) mitoribosomes 35, 36. In other ribosomes, the CP region is conventionally occupied by 5S rRNA. In addition, the mammalian mitoribosome was initially thought to contain cytosolic 5S RNA based on co-immunoprecipitation of this RNA molecule with mt-LSU [39]. Therefore, the recent cryo-EM structural studies have resolved the longstanding conundrum of whether or not mammalian mitoribosomes contain a 5S rRNA. These studies have recently been extended to show that mammalian species in general can incorporate different mt-tRNAs into the mt-LSU [40]. However, of 22 mitochondria-encoded tRNAs, only mt-tRNA^Val^ and mt-tRNA^Phe^ were found to be incorporated into the mt-LSU, and there was no evidence for tissue-specificity. Most astonishingly, however, we revealed the plasticity of this arrangement by analyzing a patient-derived cell line harboring a mutation in mt-tRNA^Val^, which results in a greatly reduced global steady-state level of mt-tRNA^Val^. Under these conditions of limiting availability of mt-tRNA^Val^, the human mitoribosome was able to switch from incorporating mt-tRNA^Val^ to mt-tRNA^Phe^. Moreover, human mitoribosomes containing mt-tRNA^Phe^ were shown to be translationally competent [40].

The substitution of 5S by a mitochondria-encoded tRNA in the mitoribosome could stem from the inability of the mammalian organelle to efficiently import RNA from the cytoplasm (discussed in [41]). The selection of mt-tRNA^Val^ and mt-tRNA^Phe^ as structural components of the mt-LSU is most likely related to the position of these genes in the mammalian mitochondrial genome. In prokaryotes, the 5S rRNA is part of a polycistronic transcript that also contains the small and large rRNAs. An analogous gene arrangement is found in most mammalian mitochondrial genomes, with mt-tRNA^Val^ and mt-tRNA^Phe^ flanking the two mt-rRNA genes. This gene arrangement in mammalian mtDNA confers a spatial advantage and stoichiometric availability of the mt-tRNA and mt-rRNA species for incorporation into the mt-LSU. However, the reason why different mt-tRNAs are found in mitoribosomes of closely related mammalian species remains to be addressed. In addition, it would be interesting to investigate the incorporation of structural RNAs into mitoribosomes in species where the arrangement of mt-rRNA and mt-tRNA on mtDNA is different from that of mammals.

### Building Mitoribosomes: New Players in the Assembly Pathway

In all systems studied thus far, ribosome biogenesis entails a complex, multistep, and tightly regulated pathway. In budding yeast, approximately 200 proteins and 70 snoRNAs have been reported to participate in the production of the cytoplasmic ribosome. Many of these proteins directly facilitate the assembly or transport of pre-ribosomal complexes from the nucleolus to the cytoplasm [42]. In bacteria, the absence of cellular compartmentalization reduces the number of proteins required for ribosome synthesis. Nonetheless, approximately 80 assembly factors, rRNA modifiers, and rRNA processing enzymes are involved in the biosynthesis of the bacterial ribosome [43]. In contrast to the relatively well-characterized ribosomal assembly pathways in bacteria and in eukaryotic cytoplasm, studies addressing the biogenesis of the mammalian mitoribosome have only recently commenced and are in their infancy [44]. We summarize below the recently discovered factors associated with the mitoribosome biogenesis process and discuss how further studies of these factors could be useful in elucidating mitoribosome assembly.

Several protein factors have thus far been identified to play roles in the assembly of the small subunit of the mitoribosome (mt-SSU) (Figure 2). Two previously mentioned 12S mt-rRNA methyltransferases, NSUN4 and TFB1M, have been implicated in mt-SSU biogenesis. NSUN4 has been shown to have a dual function: although it is required for 12S methylation, NSUN4 additionally functions in complex with MTERF4 to play a role in regulating monosome assembly, presumably by binding to the mt-LSU and facilitating interaction with the mt-SSU 14, 45. Ablation of TFB1M causes a reduction in 12S mt-rRNA steady-state level, leading to a deficiency of assembled mt-SSU [13]. ERAL1, a homolog of the bacterial Era protein, is a member of the conserved family of GTP-binding proteins and has been suggested to function as an RNA chaperone that protects 12S mt-rRNA on the mt-SSU during assembly 46, 47. In addition, the C4orf14 (NOA1) GTPase interacts with the mt-SSU, and its knockdown leads to a defect in small subunit assembly [48].

Our knowledge about the factors required for the assembly of the mt-LSU has also rapidly expanded (Figure 2). In addition to the mt-SSU, methyltransferases have also been implicated in the biogenesis of the mt-LSU. Downregulation of MRM2 or MRM3 results either in diminished levels or altered sucrose-gradient sedimentation of the mt-LSU, respectively [12]. However, it is currently not clear whether the absence of methyltransferase activity or simply the lack of the protein results in the aberrant production of mt-LSU. Two RNA-binding proteins, MTERF3 and FASTKD2, and a putative RNA helicase, DDX28, have been implicated in the assembly of the mt-LSU, although the molecular mechanisms are not understood 49, 50, 51, 52. A more pleiotropic effect on the mitoribosome was observed when the expression of RNA-binding protein GRSF1 or the putative helicase DHX30 was suppressed – this resulted in the accumulation of mt-SSU subassemblies and a decrease in mt-LSU and monosome content [49]. Similarly, a MPV17 protein family member, MPV17L2, was found to associate with the mt-LSU, but its knockdown led to instability of both the mt-LSU and mt-SSU [53]. Finally, MALSU1, a member of the ribosome silencing factor (RsfS) family [54], has been involved in assembly and/or stability of the mt-LSU 55, 56, 57. It remains to be determined whether MALSU1, similarly to its bacterial homolog, exhibits ribosome silencing activity, preventing premature association of the small and large subunits to form the monosome.

Current knowledge of the functional role of the identified mitoribosome biogenesis factors does not allow their confident assignment to particular steps in the assembly process. Without further studies, it is difficult to draw any meaningful parallels in the assembly process between the mitoribosome and its cytoplasmic or bacterial counterparts. However, a growing inventory of mitochondrial factors involved in mitoribosome production is expected to contribute to future investigations on the molecular details of its sequential assembly. Recent application of affinity purification of pre-ribosomal particles using an epitope-tagged assembly factor, combined with subsequent cryo-EM analysis, has enabled snapshots of compositionally and structurally different intermediates of budding yeast cytoplasmic ribosome biogenesis to be acquired, providing essential mechanistic details [58]. An analogous approach could be adapted for selected mitochondrial factor(s) that have already been shown to participate in mitoribosome production. Further, inactivation of specific mitoribosomal biogenesis factors can result in the accumulation of on-pathway intermediates which, again, could be analyzed by cryo-EM or proteomic profiling [59]. These approaches are likely to uncover novel assembly factors that remain attached to these incompletely assembled mitoribosomes, and could be helpful in building detailed assembly maps.

## Regulation and Coordination of Mitochondrial Gene Expression

The processes by which the basic stages of gene expression are regulated in mammalian mitochondria to adapt to metabolic demand are still not well understood. Several recent studies have brought to light the previously unknown organization of newly synthesized mtRNAs into dynamic nucleoprotein structures, the MRGs (Box 3). These subcompartments may provide spatiotemporal regulatory function for mtRNA post-transcriptional processing, allowing mt-mRNAs, mt-tRNAs and mt-rRNAs to be matured fully before release for use in protein synthesis.

Box 3 **Mitochondrial RNA Granules (MRGs)**

From their synthesis to degradation, mtRNAs undergo several transformation steps in which diverse molecular players are involved, all with the aim of enabling the faithful production of mtDNA-encoded proteins.

Maturation of mtRNAs comprises processes such as synthesis, cleavage of 5′ and 3′ ends, modification of key nucleotides, editing of 3′ ends, decoding, and eventual degradation (Box 1). The enzymes which perform these tasks are not randomly distributed in the mitochondrial matrix but are enriched in punctate structures, or foci, providing dedicated centers that participate in different stages of mtRNA metabolism.

Studies on the localization of specific mitochondrial proteins, bromouridine (BrU) incorporation, and mtDNA immunostaining have enabled the identification and characterization of foci containing nascent transcripts, called MRGs. Although found in the vicinity of mtDNA-containing foci, they are structurally distinct from nucleoids 64, 68. Diverse classes of proteins are present in MRGs. The presence of enzymes involved in the processing of mtRNAs (GRSF1 64, 68, RNase P subunits [64], and mtPAP [69]) and the post-transcriptional nucleotide modification of non-coding mtRNAs (e.g., MRM2, MRM3 [67], TFB1M, PUS1 [49], and TRMT10C [100]) led to the conceptualization of MRGs as RNA maturation centers in mitochondria.

Furthermore, because mitoribosome assembly factors (e.g., MTERF3/MTERFD1, ERAL1, DDX28, and FASTK-family proteins 49, 52) (Figure 2) and integral mitoribosome components (i.e., mt-rRNAs and mitochondrial ribosome proteins [64]) are gathered in MRGs, they have been postulated to be involved in regulating mitochondrial translation.

The last stage in the life of mtRNAs has also been proposed to take place in dedicated foci, termed D-foci [66] for its principal component, the degradosome complex. The degradosome complex is formed by mitochondrial helicase Suv3 [101] and polynucleotide phosphorylase (PNPase) 102, 103.

Although a proportion of identified D-foci also colocalize with newly synthesized RNA, similarly to MRGs, further investigation will be necessary to verify whether these foci form a subset of MRGs or are separate entities with a distinct composition and purpose.

Alt-text: Box 3

In addition, owing to the dual genetic origin of subunits of OxPhos complexes, there is a necessity for coordination of gene expression of both genomes. While this may be achieved through control of transcription and mtRNA maturation of both genomes, direct regulation of mitochondrial protein synthesis is emerging as another likely stage for ensuring correct production and assembly of OxPhos subunits. We discuss below recent breakthroughs in our understanding of how mitochondrial gene expression is coordinated.

### mtRNA Granules: A Subcompartment for RNA Maturation and Ribosome Assembly

Since first being imaged via microscopy using fluorescent (DAPI, 4′,6-diamidino-2-phenylindole) staining of DNA, mtDNA has been observed to be located in discrete punctae throughout the mitochondrial network [60]. Immunostaining experiments indicated that a range of proteins, with roles associated with mtDNA maintenance (such as packaging and replication) and transcription of the mitochondrial genome, colocalize with these mtDNA foci [61]. Together with mtDNA, these associated proteins form a nucleoprotein complex known as the mitochondrial nucleoid (Figure 1). A recent study which sought to clarify their functional organization has suggested that nucleoids are almost exclusively comprised of a single copy of mtDNA, and the compacted nucleoid structure is achieved through an interaction with mitochondrial transcription factor A (TFAM) [62].

Newly transcribed mtRNAs were found in discrete foci situated in close proximity to mitochondrial nucleoids [63]. These foci, known as MRGs (Box 3), have been found to contain numerous proteins involved in post-transcriptional processing and regulation of mtRNA, as well as factors involved in mitoribosome assembly (Figure 2) 49, 64, 65, 66, 67, 68, 69. By tracking the progress of nascent RNA transcripts through the incorporation of the uridine analog 5-bromouridine (BrU), together with the colocalization of protein factors for RNA processing 64, 68 (Boxes 1,3), these foci have come to be recognized as compartmentalized centers for RNA processing. Newly identified RNA-binding proteins were found to localize to MRGs, whose function is crucial for the activities housed within them. G-rich RNA sequence binding factor 1 (GRSF1), for example, interacts with the RNase P complex, and its loss leads to aberrant mtRNA processing [64] and a mitochondrial translation defect 64, 68. More recently, systematic approaches towards the characterization of the MRG proteome has led to the identification of further proteins involved in post-transcriptional mtRNA metabolism, including Fas-activated serine/threonine kinase (FASTK) protein family members FASTK and FASTKD5 49, 65. A further FASTK family member, FASTKD4, has also been implicated in mt-RNA processing, although this polypeptide has not yet been found to localize to MRGs [70].

In addition to MRGs, both components of the mitochondrial degradosome, RNA helicase Suv3 and 3′→5′ exoribonuclease polynucleotide phosphorylase (PNPase), have been found to localize in discrete punctae known as D-foci [66]. A portion of these identified D-foci colocalize with newly synthesized, BrU-labeled mtRNAs, and therefore potentially overlap spatially and functionally with MRGs (Figure 1). These D-foci, therefore, may contain specific mtRNAs that require the action of the degradosome for their maturation, for example ND6 [65], or may represent turnover events of newly synthesized but misprocessed RNAs.

Many factors necessary for mitoribosome biogenesis have also been localized in MRGs (Figure 2). Hence, it has been proposed that MRGs are also centers for mitoribosome biogenesis, with a function analogous to that of the nucleolus where initial steps of cytoplasmic ribosome assembly are performed 49, 52, 71. Fractionation-based enrichment experiments suggested that nucleoids copurify with mitochondrial factors involved in mtRNA metabolism, mitoribosome biogenesis, and translation, as well as with mitochondrial ribosomal proteins (MRPs). The latter have also been found to reside in MRGs 48, 72, 73. It has been suggested that early steps of mitoribosome assembly take place co-transcriptionally, with incorporation and assembly of MRPs occurring in concert with mt-rRNA nucleotide modifications 74, 75. This overlap in the protein complement associated with either the mitochondrial nucleoid or MRGs suggests intimate association of these two entities. Therefore, it is possible that both mtDNA and its transcription products are partitioned within non-membrane compartments to provide a greater degree of spatiotemporal regulation of mtRNA processing, for example, by sequestering immature mtRNAs within MRGs, away from mitochondrial translation processes [76] (Figure 1).

### Coordination of nDNA and mtDNA Gene Expression

Owing to the dual genetic origin of OxPhos complexes, there is a need for tight coordination between the synthesis of nucleus-encoded and mtDNA-derived components to prevent the accumulation of unaffiliated subunits in the mitochondrial membrane. In the past this problem was investigated mainly in the context of transcriptional response by the nucleus-encoded mitochondrial genes (e.g., [77]). However, whether the translation of nucleus-encoded and mitochondria-encoded transcripts change concordantly was not clear. A recent study in yeast has sought to address the mechanisms responsible for controlling the mitochondrial–nuclear protein balance using whole-cell genomic profiling approaches upon switching from anaerobic to oxygen-dependent respiration [78]. In response to nutrient shift, the nucleus-encoded OxPhos mRNAs are induced rapidly, with mitochondria-encoded OxPhos messages being upregulated much more slowly. However, yeast ribosome profiling experiments revealed that, during adaptation from anaerobic to aerobic growth, mitochondrial and cytosolic translation are regulated in a rapid and coordinated manner. Furthermore, this adaptation is unidirectional because mitochondrial gene expression was dependent on cytosolic translation and the import of nucleus-encoded factors into mitochondria, whereas inhibition of mitochondrial protein synthesis had no effect on the translation efficiency of nucleus-encoded OxPhos subunits. In yeast, many transcript-specific activators, that have roles in initiation and\or elongation, control the translation of mitochondrial mRNAs [79]. Unraveling the roles of these translation activators in mediating the synchronized cytoplasmic–mitochondrial response will be an important goal for future research.

There are significant differences, however, between the genetic systems of yeast and mammalian mitochondria, and it will be necessary to determine whether the transcription/translation control observed in yeast during adaptation of OxPhos to different metabolic settings is a universal process. For instance, the structures of mammalian mt-mRNAs differ significantly from those of their yeast counterparts (the lack of introns, very short or absent untranslated regions, or the presence of polyadenylation in mammalian mRNAs). In addition, only one mitochondrial translation activator has been identified in mammalian mitochondria 80, 81, suggesting the mechanisms of mammalian mitochondrial gene regulation have diverged significantly from those found in yeast. For these reasons, the application of similar whole-cell genome approaches to mammalian systems would be advantageous.

Feedback mechanisms that fine-tune the translation of a subset of mitochondria-encoded proteins to optimize the levels of assembled into OxPhos complexes have been a subject of intense research in yeast 82, 83. By contrast, much less is known about these processes during the biogenesis of the mammalian OxPhos system. A very recent study has identified ‘mitochondrial translational plasticity’ as a means to coordinate the assembly of cytochrome *c* oxidase (COX, complex IV) with mitochondrial translation stalling and restarting according to the availability of nucleus-encoded subunits [84]. The authors found that, in the absence of COX4 (the first incorporated nucleus-encoded COX subunit), translation of mtDNA-encoded COX1 is halted, resulting in stalled ribosomes carrying nascent COX1 peptides associated with the early COX assembly intermediates [84]. This represents a novel pathway of mitochondrial translational control whereby lack of an available nucleus-encoded OxPhos subunit binding partner acts as a direct signal to inhibit mitochondrial translation, preventing the accumulation of an excess of mtDNA-derived subunits that are unaffiliated to OxPhos complexes.

It will be intriguing to determine, through applying a similar experimental approach, whether there is related translational plasticity during the assembly of the other OxPhos complexes with dual genetic origin (complexes I, III, and ATP synthase).

## Concluding Remarks and Future Perspectives

Recent years have brought many important insights into the regulation of mtDNA gene expression. Several novel factors have been identified, either via basic research approaches, or through the study of patients with respiratory chain disorders. The characterization of these factors has furthered the basic understanding of their function in the context of mtRNA metabolism and translation, in addition to shedding new light upon these processes. Nonetheless, many fundamental questions, frequently related to the pathology of mitochondrial diseases, remain unanswered and new questions have arisen (see Outstanding Questions). The future research of mitochondrial gene expression is expected to continue focusing on the regulatory function of post-transcriptional mtRNA modifications as well as on the biogenesis of the mitoribosome and of RNA granules. The application of genome-wide approaches to study mtDNA gene expression, such as ribosome profiling, is expected to be more widespread as a tool to investigate the basic features and pathology-related defects of mitochondrial protein synthesis. Studies of the molecular mechanisms that govern mtDNA gene expression are impeded by the inability to manipulate the mitochondrial genome at will, hence a routine analysis of the role of *cis*-acting elements involved in mitochondrial transcription or RNA maturation is currently impossible [85]. Therefore, future work should also focus on resolving this practical issue to enable a reverse genetics approach for a comprehensive structure–function study of mtDNA gene expression. Finally, the development of an *in vitro* system for mitochondrial translation would be useful to directly establish the mechanistic details of intra-mitochondrial protein synthesis. This task is currently confounded, however, by our incomplete understanding of the basic components required and the need for membrane association of the mitochondrial translation machinery.

Outstanding Questions What further factors are involved in post-transcriptional mtRNA modifications?

How is processing at non-canonical cleavage sites in polycistronic mtRNA transcripts achieved?

What are the precise roles of mtRNA nucleotide modifications in mitochondrial ribosome biogenesis and/or translation regulation?

How is assembly of mitochondrial ribosomal proteins into the large and small mitoribosomal subunits regulated?

What functional roles do MRGs play in the spatial and temporal organization of mtRNA processing?

What signals/mechanisms are required for concerted translation of cytoplasmic and mitochondrial OxPhos mRNAs?
